# Choroidal Vascularity Index (CVI) - A Novel Optical Coherence Tomography Parameter for Monitoring Patients with Panuveitis?

**DOI:** 10.1371/journal.pone.0146344

**Published:** 2016-01-11

**Authors:** Rupesh Agrawal, Mohammed Salman, Kara-Anne Tan, Michael Karampelas, Dawn A. Sim, Pearse A. Keane, Carlos Pavesio

**Affiliations:** 1 Moorfields Eye Hospital, NHS Foundation Trust, London, United Kingdom; 2 University College London, Institute of Ophthalmology, London, United Kingdom; 3 National Healthcare Group Eye Institute, Tan Tock Seng Hospital, Singapore, Singapore; 4 Yong Loo Lin School of Medicine, National University of Singapore, Singapore, Singapore; 5 West Hertfordshire NHS Trust, London, United Kingdom; 6 NIHR Biomedical Research Centre, Moorfields Eye Hospital, London, United Kingdom; Charité University Medicine Berlin, GERMANY

## Abstract

**Purpose:**

To compute choroidal vascularity index (CVI) using an image binarization tool on enhanced depth imaging (EDI)-optical coherence tomography (OCT) scans as a non-invasive optical tool to monitor progression in panuveitis and to investigate the utility of volumetric data from EDI-OCT scans using custom image analysis software.

**Materials and Methods:**

In this retrospective cohort study, segmented EDI-OCT scans of both eyes in 19 patients with panuveitis were taken at baseline and at 3-month follow-up and were compared with EDI-OCT scans of normal eyes. Subfoveal choroidal area was segmented into luminal (LA) and stromal interstitial area (SA). Choroidal vascularity index (CVI) was defined as the proportion of LA to the total circumscribed subfoveal choroidal area (TCA).

**Results:**

The mean choroidal thickness was 265.5±100.1μm at baseline and 278.4±102.6μm at 3 months follow up (p = 0.06). There was no statistically significant difference in TCA between study and control eyes (p = 0.08). CVI in the control group was 66.9±1.5% at baseline and 66.4±1.5% at follow up. CVI was 74.1±4.7% at baseline and 69.4±4.8% at 3 months follow up for uveitic eyes (p<0.001). The % change in CVI was 6.2 ±3.8 (4.3 to 8.0) for uveitic eyes, which was significantly higher from % change in CVI for control eyes (0.7±1.1, 0.2 to 1.3, p<0.001).

**Conclusion:**

The study reports composite OCT-derived parameters and CVI as a possible novel tool in monitoring progression in panuveitis. CVI may be further validated in larger studies as a novel optical tool to quantify choroidal vascular status.

## Introduction

Panuveitis is a heterogenous group of inflammatory disorders involving the posterior segment of the eye, characterized by inflammation of the retina, choroid, and vitreous, with or without a systemic associated inflammatory process.[1] It is reported in 10–15% of patients with uveitis.[2–4]

The presence of tools to monitor disease progression in patients on potentially life-threatening drugs for the treatment of panuveitis is currently limited. More often than not, these patients require systemic immunosuppressive therapy, and there is no consensus amongst physicians about the duration of therapy.[5,6] In the absence of a reliable method to monitor disease activity patients may end up under or over-treated, which is especially worrying when potentially dangerous immunosuppressive drugs are used, exposing patients to unnecessary risks.[7] The imaging modalities used in the diagnosis and follow up of uveitis are fundus fluorescein (FFA) and indocyanine green angiography (ICGA),[8] optical coherence tomography (OCT),[9] wide field retinal imaging[10] and fundus autofluorescence.[11] Functional tests, such as visual fields, and electrophysiological tests, also have a role in the diagnosis and management of panuveitis.

Angiography demonstrates different patterns of fluorescence providing indication about disease activity, however, the presence of leakage and staining, which is useful in monitoring disease progression, however they are invasive in nature and not easily used for routine follow-up.[7] The advent of OCT and autofluorescence has allowed more in-depth analysis of the vitreous-retina-choroid in a non-invasive way.[12] OCT can provide real time optical cross-sections of the retina, retinal pigment epithelium (RPE) and choroid. Further advances in technology on enhanced depth imaging (EDI)–OCT scans, have provided more insight into the structural changes allowing for quantitative measurements of choroidal vasculature in patients with panuveitis.[13–23]

Numerous studies have been published regarding choroidal thickness (CT) as the denominator for disease activity. However CT may not be a robust tool in clinical research because there are many physiological factors such as diurnal variation, refractive error, gender, and age that affect it.[21] Moreover, we do not know which structures within the choroid exhibit changes in uveitis. Measures of CT may be helpful but, we need to explore and measure novel indices in the choroid to be used as indicators of ocular and systemic health. In this report, we envisage to investigate the application of semi-automated software as a possible optical tool in patients with varying forms of posterior or panuveitis.

## Materials and Methods

After obtaining research and ethics committee approval (ROAD14/002) for retrospective clinical data and image analysis from Hospital Ethics Committee at Moorfields Eye Hospital, images and data of the patients with panuveitis who had been followed up with EDI-OCT scans were used for this longitudinal study. Patient records/information were anonymized and de-identified prior to analysis. The study was conducted as per the tenets set forth in the Declaration of Helsinki.

EDI-OCT images from patients with panuveitis attending a specialist uveitis clinic (CP) over a period of one year were included in the study. EDI-OCT scans from normal eyes were used as controls. The following information was retrieved in all the patients enrolled in this study: demographics, best corrected Snellen’s visual acuity (was converted to logMAR (logarithm of the minimum angle of resolution) units for statistical analysis, disease activity, baseline EDI-OCT scans, follow up EDI-OCT scans and any other ancillary investigations. EDI OCT scans at baseline (at time of first EDI OCT scan, with or without treatment) and at 3 months follow up was used for further analysis. Consent was not obtained from the patients since this was a retrospective study with no patient identifiers from the data.

OCT images were obtained using the Spectralis OCT device (Spectralis; Heidelberg Engineering; Heidelberg, Germany) by experienced ophthalmic technicians. All the images were obtained using the EDI protocol first described by Spaide et al.[12] In brief, the OCT device was positioned in close proximity to the patient’s eye in order to acquire an inverted image, with 7 equally-spaced OCT B-scan sections obtained in a 20° x 15° horizontal raster pattern. Volumetric data from EDI-OCT scans were computed using custom image analysis software (OCTOR, Doheny Image Reading Center, Los Angeles, Fiji; http://imagej.nih.gov/ij, version 2.0.0-rc.15/1.49j, date: 2014-10-06).[24]

### Volume analysis of total choroid from EDI-OCT Images

**Fig 1:**
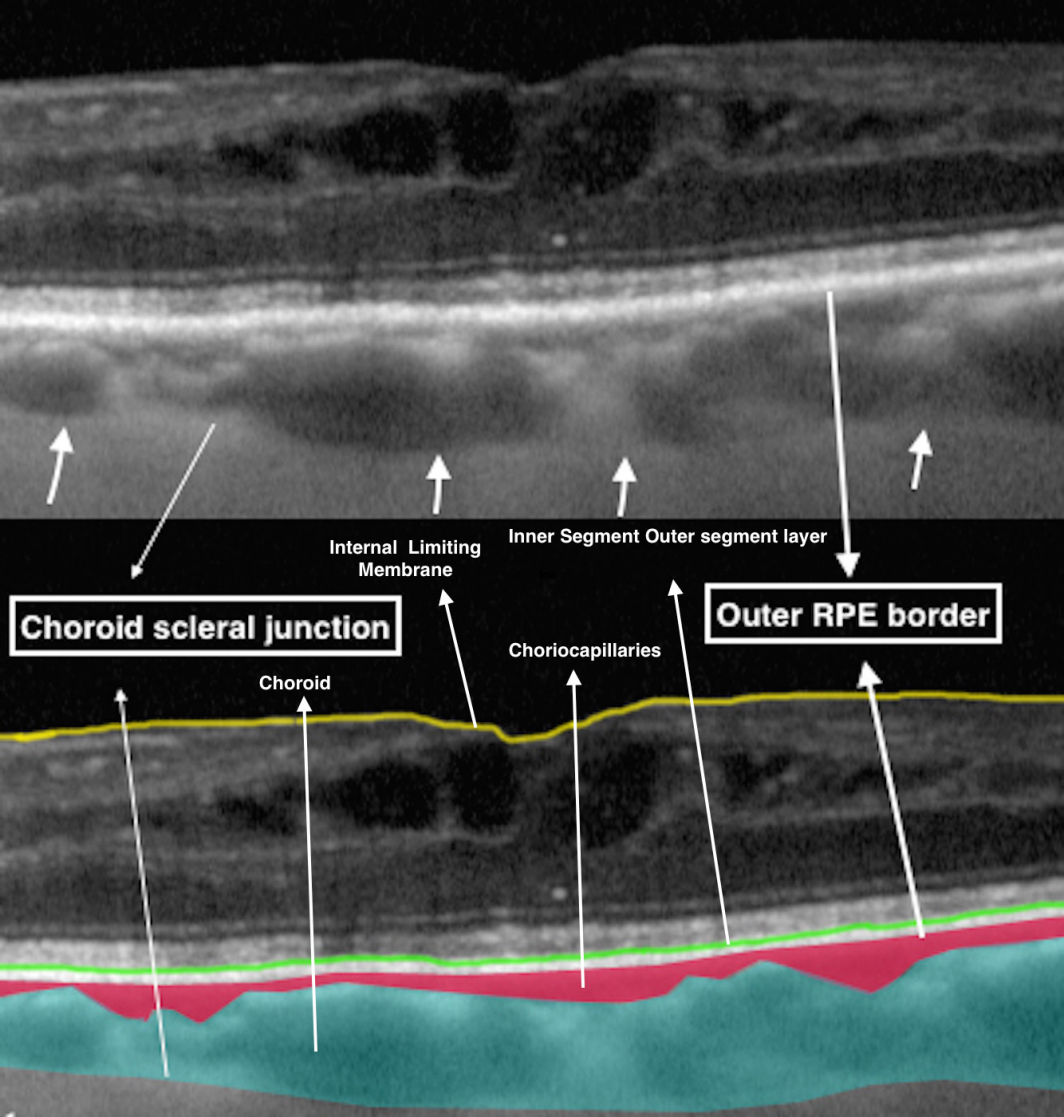
Enhanced depth imaging (EDI) scan from spectral domain optical coherence tomography (SD OCT) with semiautomated software image segmentation of the choroid representing retinal pigment epithelium (RPE) and choroid scleral junction as the boundary for choroidal area. *Definitions of Areas on EDI-OCT*: ***Subfoveal choroidal thickness (SFCT)*:** Distance between the outer border of hyperreflective retinal pigment epithelium (RPE) and the outer border of the choroid beneath the centre of the fovea. ***Luminal Area (LA)*:** Area of dark pixels in the choroid seen on binarised EDI-OCT images was defined as LA or vascular area. This area corresponds to the vascular area of the choroid when compared to original EDI-OCT images. ***Stromal Area (SA)*:** Area of light pixels in the choroid seen on binarised EDI-OCT images was defined as SA or interstitial area. This area corresponds to the interstitial areas of the choroid when compared to original EDI-OCT images. ***Choroidal Vascularity Index (CVI)*:** The ratio or proportion of the LA within the circumscribed subfoveal choroidal area.

Quantitative analysis was performed by one of the authors (MS) using the semi-automated OCTOR custom image analysis software. The software has been described and validated in previous reports but is currently not available for public download.[25–27] Raw OCT data were exported using.xml format from the Heidelberg Spectralis OCT system and imported into the OCTOR software and files were converted to the OCTOR file format, termed ‘OCTOS’. For each OCT image set, inner and outer boundaries for the retina, retinal pigment epithelium (RPE) complex and the choroid were manually segmented **(Fig 1).** All boundaries were manually drawn in accordance with the currently described OCT landmarks in the International Nomenclature for OCT (IN-OCT) meeting consensus for normal OCT terminology.^17^ As the manual segmentation was complex, only 11 out of 19 study eyes were segmented. Volume (mm^3^), thickness (μm) and intensity were automatically computed for retina, RPE and choroid at both baseline and 3-month follow up for both study and normal eyes using all seven raster scans using automated software tool of OCTOR.

### Binarization of subfoveal choroidal EDI-OCT images

The central scan passing through the foveal/subfoveal region was selected and one image per patient per visit was used for analysis. The protocol proposed by Sonada et al[28] was used with minor modifications. The binarization of the subfoveal choroidal area was performed by one of the authors (RA). Under this protocol–images were analysed using public domain software Fiji. [24]

The detailed image analysis algorithm is provided in **S1 Appendix**. In brief, after uploading the images on Fiji, the images were converted to 8-bit images to allow the application of auto-threshold. The scale was set by converting pixels to microns using the horizontal scale present on the images from SD-OCT scans, so that a 1500μm linear line centred at fovea could be drawn. The choroidal block beneath the line was segmented and total subfoveal choroidal area (TCA) was computed. Brightness was reduced to clearly visualise the choroidal vessels and also to minimize the noise in the EDI–OCT images.

**Fig 2:**
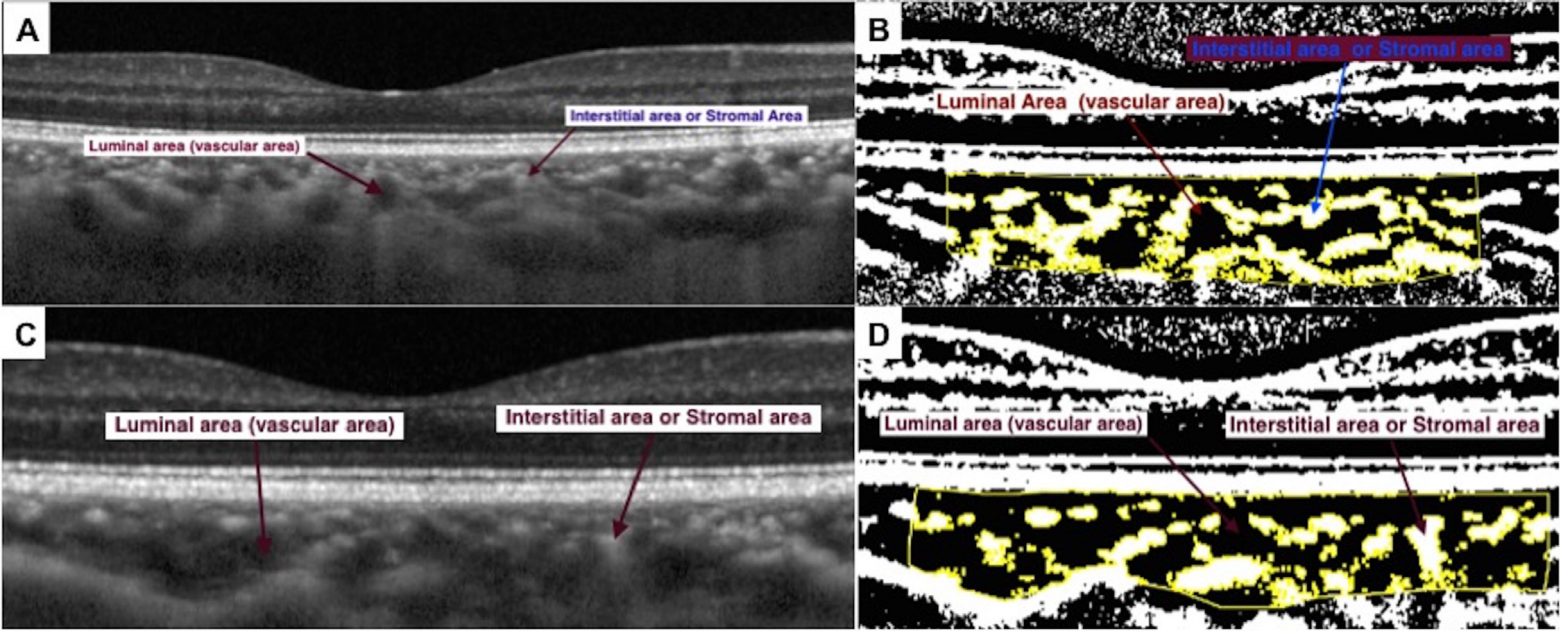
Choroidal enhanced depth imaging (EDI) SD OCT scans showing black areas (luminal areas, LA) (2A and 2C) and white areas as interstitial areas (stromal area, SA) (2A and 2C). The areas are seen more clearly after image segmentation using semiautomated software (2B and 2D).

Auto local threshold was applied using different methods (Otsu, Phasalkar, Niblack) but Niblack auto local threshold was found to give best resolution and demarcation of luminal (vascular) (LA) and stromal (interstitial) area (SA). This is also in accordance with the earlier published protocol by Sonada et al.[28] Hence, Niblack auto local threshold was applied in all our images to compute LA and SA in mm^2^. The image adjusted by auto local threshold was again converted back to RGB (red, green, blue) image and the LA and SA was determined using the colour threshold tool (**Fig 2**).

**Fig 3:**
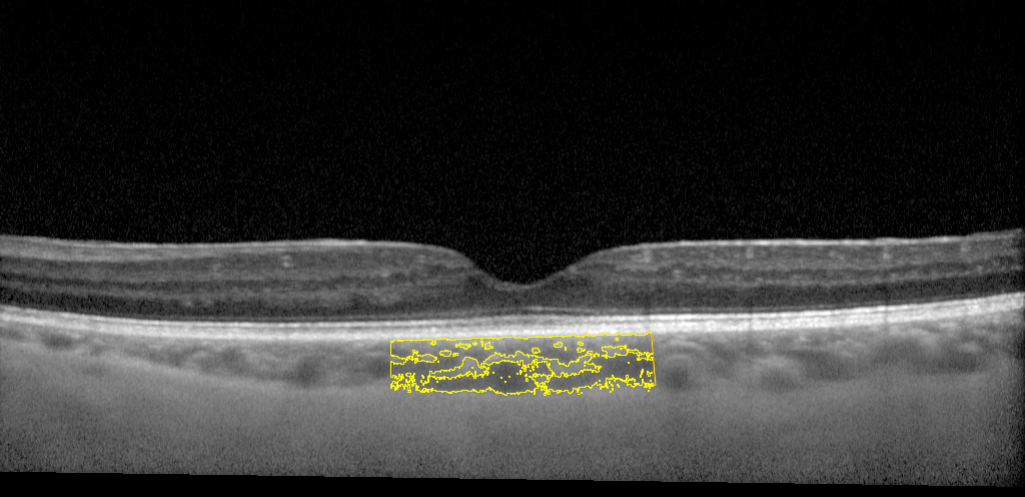
A schematic illustration of the EDI SD OCT scan with superimposed binarized image showing segmentation of the choroidal structures.

Choroidal vascularity index (CVI), defined as the proportion of LA to TCA, was computed for all the images at baseline and follow up (**Fig 2**). **Fig 3**represents the superimposed image of the binarized segment over the conventional EDI OCT scan, and demonstrates the LA and SA in the optically segmented block of choroidal tissue.

### Statistical analysis

Visual acuity was converted to logMAR and its correlation with quantitative measurements of retinal, RPE, choroidal thickness and volume was done using t-test. Quantitative measurements at two separate visits 3 months apart were statistically analysed using Wilcoxon signrank test and a *p* value of <0.05 was considered statistically significant. Using the t-test and by comparing choroidal and retinal thickness with the fellow normal eyes, the correlation between study eyes (study group) and normal eyes (control group) was calculated. The ANOVA test was also used to compare the variance in means at different time points (two time points in this study), and also to analyse variance between study eyes and control eyes. Statistical analysis was performed using commercially available software (Intercooled STATA for Windows Version 13; Stata Corp). Data is presented in **S2 Appendix.**

## Results

19 eyes from 19 patients with posterior or panuveitis were included as study eyes with normal eyes as control group. The mean age was 51.5 years (range 23.00 to 76.00) with 10 (52.63%) males and 9 (47.37%) females. The mean BCVA for study eyes was 0.26 logMAR (range 0.00 to 1.20, Snellen visual acuity 6/12). There were 3 (15%) patients with an epiretinal membrane and 4 (20%) patients with cystoid macular edema. BCVA in these eyes was significantly lower compared to the rest of the eyes (p = 0.006). No significant correlations were observed between the retinal measures and logMAR VA or vitreous cellularity (p = 0.15). All the patients had EDI-OCT scans at baseline and at 3-month follow up. However, only five out of 19 patients had EDI OCT scans at the 6 and 9-month follow up and hence the measurements from 6 and 9-month follow up scans were not included for analysis.

### Retinal and choroidal thickness, volume and intensity measurements using OCTOR software for study eyes

The mean retinal thickness was 330.7±173.1μm at baseline and 303.1±111.3μm at 3-month follow up (p = 0.65). The mean CT was 265.5±100.1μm at baseline and 278.4±102.6μm at 3-month follow up (p = 0.06). There was no correlation with age, gender or visual acuity for any of the measurements. Changes in intensity measurements for retinal, RPE and choroidal tissue are also presented in **Table 1.** There were no statistically significant changes in the measurements between baseline and follow up.

**Table 1:** Retinal, retinal pigment epithelium (RPE) and choroidal thickness and intensity measurement for uveitic eyes using OCTOR software at baseline and presentation.

Foveal central subfield–entire scan	Baseline	3 months	P
	Mean ± SD (Range)		
**Retinal thickness**	330.7±173.1(216.6 to 833.4)	303.1±111.3 (210.3 to 613.8)	0.65
**RPE thickness**	31.7 ± 6.6 (21.2 to 46.6)	32.7 ± 6.7 (22.6 to 45.6)	0.79
**Choroidal thickness**	265.5 ± 100.1 (148.9 to 514.6)	278.4 ± 102.6 (168.7 to 536.2)	0.06
**Retinal intensity**	0.3 ± 0.1 (0.2 to 0.4)	0.3 ± 0.1 (0.1 to 0.4)	0.47
**RPE intensity**	0.8 ± 0.0 (0.8 to 0.9)	0.8 ± 0.0 (0.7 to 0.9)	0.06
**Choroidal intensity**	0.4 ± 0.1(0.3 to 0.6)	0.4 ± 0.1(0.3 to 0.5)	0.53

**RPE:** Retinal Pigment Epithelium; **SD:** Standard Deviation, Units for thickness measurements: **μm**,

### Image binarization tool was applied to compute subfoveal TCA, LA, SA, LA/SA and CVI

**Fig 4:**
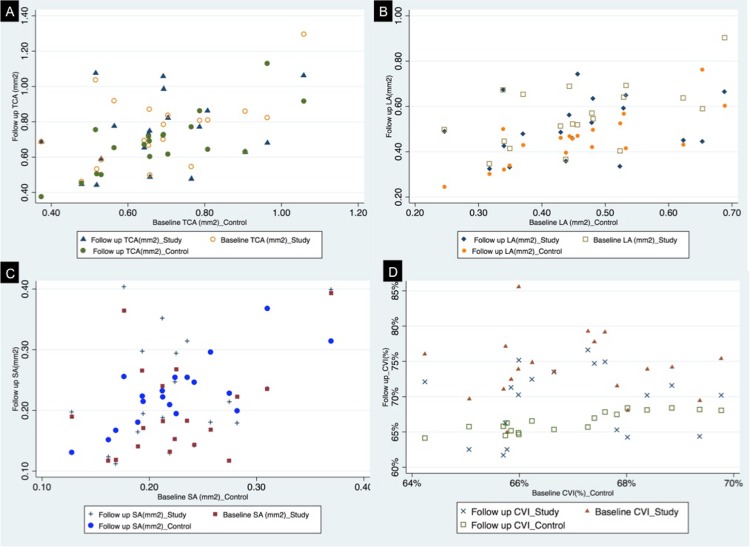
**4A:** Scatter plot representing the total subfoveal choroidal area (TCA) for uveitic eyes and control eyes at baseline and 3 month follow up. 4B: Scatter plot representing the luminal area (LA) for uveitic eyes and control eyes at baseline and 3 month follow up. **4C:** Scatter plot representing the stromal area (SA) for uveitic eyes and control eyes at baseline and 3 month follow up. **4D:** Scatter representing the choroidal vascularity index (CVI) for study (uveitic) eyes and control eyes at baseline and 3 month follow up.

**Fig 5:**
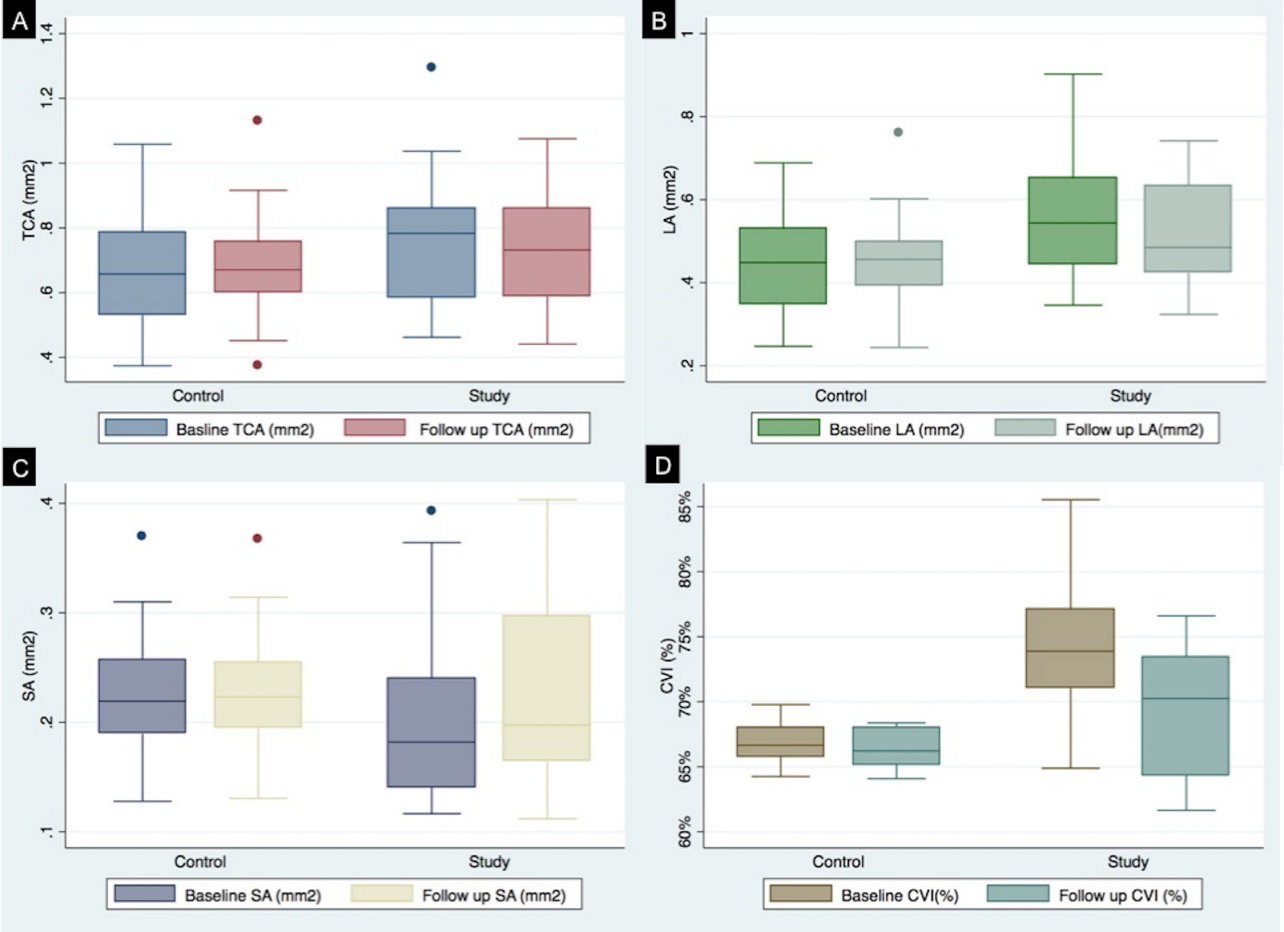
Composite box plots representing the baseline and follow up measurements for total choroidal area (TCA) (A), luminal area (LA) (B), stromal area (SA) (C) and choroidal vascularity index (CVI) (D) for both control and uveitic (study) eyes.

There was no statistical difference between baseline and follow up values for TCA, LA, SA for the control group (Table 2). **Fig 4A** demonstrates TCA for both control and study eyes at baseline and at follow up. **Fig 4B** represents LA for both study and normal eyes at baseline and follow up. **Fig 4C** gives comparative analysis of SA at baseline and follow up for both study and control eyes. CVI in the control group was not statistically affected at baseline (66.9±1.5%) and follow up (66.4± 1.5%). **Fig 4D** represents a comparative plot for CVI for study and control group. LA/SA was significantly different at baseline and follow up for control eyes (p = 0.006). TCA did not show any statistically significant difference at baseline and follow up for uveitic eyes. LA, however, was significantly different at baseline (0.6±0.1) and follow up (0.5± 0.1) (p = 0.01). There was associated increase in SA from 0.2±0.02 at baseline to 0.23 ± 0.09 at 3 months follow up (p = 0.005). CVI in the uveitic eyes was 74.1±4.7% at baseline, and was reduced to 69.4±4.8% (p<0.001) at follow up. LA/SA also showed statistically significant difference between baseline (3.0±0.9) and follow up (2.3±0.5). **Fig 5**presents a composite box plot for TCA, LA and SA for both study and control eyes at baseline and follow up.

**Table 2:** Image binarization indices for both study and control eyes at baseline and at three months follow up.

1500μm of foveal central subfield area using image binarization	Baseline	3 months	P
	Mean ± Standard deviation (Range)		
**Total subfoveal choroidal area (TCA), mm**^**2**^			
**Control eyes**	0.7± 0.2 (0.4 to 1.1)	0.7 ± 0.2(0.4 to 1.1)	0.79
**Study eyes**	0.8 ± 0.2 (0.5 to 1.3)	0.7 ± 0.2 (0.4 to 1.1)	0.20
**Luminal area or vascular area (LA), mm**^**2**^			
**Control eyes**	0.5 ± 0.1 (0.2 to 0.7)	0.4± 0.1 (0.2 to 0.8)	0.90
**Study eyes**	0.6 ± 0.1 (0.3 to 0.9)	0.5± 0.1 (0.3 to 0.7)	0.01
**Stromal area or Interstitial area (SA), mm**^**2**^			
**Control Eyes**	0.2 ± 0.6 (0.1 to 0.4)	0.3 ± 0.6 (0.1 to 0.4)	0.60
**Study Eyes**	0.2 ± 0.2 (0.1 to 0.4)	0.2 ± 0.1 (0.1 to 0.4)	0.05
**Choroidal vascularity Index (LA/ TCA) (CVI)**			
**Control eyes**	66.9±1.5 (64.2 to 69.8)	66.4± 1.5(64.1 to 68.4)	0.06
**Study eyes**	74.1±4.7(64.9 to 85.5)	69.4±4.8(61.6 to 76.6)	<0.001
**Luminal/ Stromal (LA/ SA)**			
**Control eyes**	2.0±0.1(1.8–2.3)	2.0±0.1(1.8–2.2)	0.06
**Study eyes**	3.00±0.9(1.8–5.9)	2.3±0.5(1.6–3.3)	<0.005

### Comparison between uveitic and control eyes

There was no statistically significant difference in TCA between uveitic and control eyes (p = 0.08). One-way ANOVA analysis was also performed to analyse the difference between means. TCA between uveitic and control eyes did not show statistically significant difference at baseline (R^2^ = 0.04, p = 0.22) and at 3 months (R^2^ = 0.02, p = 0.38). LA showed statistically significant variation between the study and control eyes on ANOVA analysis (R^2^ = 0.14, p = 0.02). There was no statistically significant difference in SA between uveitic and study eyes. (R^2^ = 0.03, p = 0.26). The most striking difference was seen in the CVI of the study and control groups (R^2^ = 0.52, p<0.001). The CVI in study group showed a statistically significant increase of 3.7% (p<0.001, Wilcoxon signed-rank test). At 3 months follow up, CVI between control and study eyes showed a statistically significant difference of 2% (p = 0.04, t test). Also, on ANOVA analysis, statistically significant difference was noted between CVI for two groups at 3 month follow up. (R^2^ = 0.16, p = 0.01). The comparative scatter plot (**Fig 4D**) represents the CVI for both uveitic and control eyes at baseline and follow-up, demonstrating minimal change in CVI for control eyes as against significant variation in the CVI for the uveitic eyes.

### Change in TCA, LA, SA and CVI

We also computed percentage (%) change in the CT, TCA, LA, SA and CVI at 3months from baseline readings. % change was computed by dividing the difference between baseline and follow up values for CT, TCA, LA, SA and CVI by the respective original (baseline) values for all the parameters and multiplying that factor by 100. The % change in CVI was 6.2 ± 3.8 (4.3 to 8.0) for study group, which was significantly higher than % change in CVI for control eyes (0.7 ±1.1, 0. 2 to 1.3, p<0.001). % change in CT, TCA, SA and LA was not statistically significant.

## Discussion

Due to the heterogeneity of the choroid and its fenestrated vascular structure, segmentation of the choroid in different layers is a daunting task. Although some customized software may be used for automated choroidal image segmentation[22,23], further refinement and generalizability is warranted for wider application of this technology. We have demonstrated relatively stable CVI but a significant variation in CT, TCA, LA, SA and LA/SA between visits and a significantly different CVI between uveitic and control eyes with non-significant difference in CT.

Several attempts have been made to unfold the mystery of the typical angio-architecture of choroid, both by *in vitro* and *in vivo* tools, in normal and diseased choroid.[9,12–15,28–40] *In vitro* techniques, such as routine histology using fixatives, intravascular casting using neoprene latex and methyl methacrylate, scanning electron microscopy, flat mount preparations with or without immunohistochemical staining, have failed to fill the gap in our understanding of choroidal vasculature and of the exact proportion of vascularity (vessels: stromal ratio) in normal choroid.[41,42] With significant advances in technology, we are now able to image the choroid, and there is an increasing amount of literature being published on the choroid and its association with local or systemic disease, thus enhancing our understanding of choroidal structure and its dynamics in different disease process.

ICGA is the current gold standard modality for choroidal pathology and vascularity, however due to its invasive nature, it is less preferred as a monitoring tool.[43] In addition, ICGA does not allow cross-sectional analysis of the choroid, and the mechanism and quantification for hypofluorescence lesions is still under investigation.[44]

OCT has been universally adopted as an essential tool in management and follow up of uveitis.[32,38,39,45,46] Many attempts have been made to examine retinal, vitreoretinal and choroidal features using this non-invasive modality.[9,14,15,17] EDI OCT has revolutionised the way to look at the characteristic features in choroid in patients with retinal diseases and uveitis.[12,28,37,39,47–50] Imaging the choroid using OCT was not possible a few years ago due to the attenuation of the light signal by the retinal pigment epithelium (RPE). Repositioning the OCT closer to the eye resulted in an inverted mirror image, with the choroid moving closer to the zero delay line, replacing vitreous, thus resulting in very high resolution imaging of the choroid on EDI-OCT scans.[12] The choroidal thickness reported in various OCT derived studies in healthy population ranges from 262–332μm.[51–53] In the current longitudinal study, we investigated two different semi-automated software applications to quantify and characterize the choroidal changes in patients with panuveitis.

Karampelas et al. have previously published the choroidal features in patients with idiopathic uveitis[14] using OCTOR software.[25] The similar novel segmentation protocol using OCTOR software was applied on a longitudinal dataset of EDI OCT images in patients with heterogenous forms of posterior or panuveitis. There was significant difference in choroidal thickness between the normal eyes and uveitic eyes. However, on 3-month follow up EDI OCT scans, the choroidal thickness did not show any significant difference in the study subjects. We also analysed the choroidal volume and intensity of RPE and choroid, and there was statistically significant difference between baseline and follow up images.

Though OCT image segmentation is very precise and provides demarcating lines for both retina and choroid, it often requires significant time to segment the images, limiting the widespread use of this novel tool. Also, on OCTOR software, even though we were able to segment the choroidal layers into Sattler’s and Haller’s layer, we could not differentiate choroidal angioarchitecture.

Branchini et al. were first to publish OCT analysis of vasculature and light/dark ratio using customized software based on MATLAB.[54] They used Otsu’s method to reduce intra-class variance. The authors reported a mean light/dark ratio of 0.271±0.08. They also computed the mean ratio of the large choroidal vessel layer to the total choroidal thickness as 0.7±0.06.[54] The authors hence demonstrated rich vascularity of the choroid which was similar to the report published by Sohrab et al.[55] wherein the authors reported mean vessel density of approximately 87% (79–93%) within the outer part of the choroid.

In our current study, we further investigated the image binarization tool as proposed by Sonoda et al.[28] for computing CVI. There is significant variation in CT with age, refractive error, time of the day the scans are done, intraocular pressure and other confounding factors. Despite this, we hypothesise that CVI is less likely to be affected, thus constituting a more robust and consistent biomarker than CT. Sonoda et al. have published that the vascularity proportion of choroid (as defined by LA/TCA) is 65% in normal population with good inter-observer and intra-observer agreement.[28] They have also demonstrated the statistically significant changes in the vascular proportion of the choroid in AMD patients following photodynamic therapy (PDT).[28]

As demonstrated by Sonoda et al.,[56] the mean total cross sectional choroidal area for the entire length of EDI-OCT scan was 1.84mm^2^ with luminal area constituting 1.21mm^2^ and stromal area constituting 0.63mm^2^. When there was a change in TCA, the LA/SA calculated by the authors determined if the increase/decrease was due to a greater increase/decrease of luminal or stromal area. For example, TCA and the LA/SA decreased significantly with increasing age, indicating that LA decreased more than SA with increasing age. However, computing the CVI (LA/TCA) provided a more stable and consistent biomarker. The proportion of vascularity within the choroid, previously demonstrated by Sonoda et al. in an earlier report, is approximately 65% in normal controls.[28] The CVI calculated using the mean values of LA and TCA from the later report by Sonoda et al., is 65.76%.[56] This has not been calculated in the report published by the authors.[56]

In our study, by measuring a novel parameter, CVI, we were able to determine if there was an increase or decrease in vascularity. We have demonstrated little if any variation in the CVI at baseline and at follow up for control eyes, compared to a significant drift in the CVI between visits for the uveitic eyes. The change in the vasculature possibly affects the thickness of the choroid, which is used as a tool to monitor disease progression in choroidal disease.

In this study, applicability of CVI as a parameter or tool for disease monitoring in a subgroup of patients with panuveitis was tested. As seen in scatter plot (**Fig 4D**), the CVI is relatively consistent at baseline and at follow up in control eyes but is comparatively more variable in patients with uveitis. Calculating the CVI and % change in CVI would provide us with information on the proportion of vascularity in the choroid. This vascularity index can hence be used as an optical biomarker of disease activity and possibly choroidal perfusion status.

Our study has several limitations such as a small sample size as well as the fact that inter-observer agreement and inter-session agreement were not performed. Nevertheless it has already been validated in previous reports from Sonoda et al.[28,56]. Also, in this study we have not compared this tool with other inflammatory biomarkers such as vitreous haze.

In conclusion, we report composite OCT-derived parameters and CVI as a possible novel tool in monitoring progression in posterior or panuveitis. Further larger population based cross sectional and longitudinal comparative studies are warranted to validate the application of CVI as a robust and reliable biomarker for diseases affecting choroid.

## Supporting Information

S1 Appendix The image binarization protocol with the corresponding images at each step of image segmentation. (PDF)

S2 Appendix The dataset for the study in excel spreadsheet format. (XLSX)
