# Supplementary material for: Choroidal Vascularity Index (CVI) - A Novel Optical Coherence Tomography Parameter for Monitoring Patients with Panuveitis?
Source: PLoS One. 2016 Jan 11;11(1):e0146344. doi: 10.1371/journal.pone.0146344 (PMC4713828; doi:10.1371/journal.pone.0146344)
Supplement: S1 Appendix — (PDF) [file pone.0146344.s001.pdf]

Methodology

Firstly, the 1x1 pixel image of the OCT was opened in ImageJ, and the scale was set. The line tool was used to measure the pixel length of 200 um as given in the scale at the bottom of the OCT scan image (Fig, 1). To set the scale, the option Analyse > Set Scale was selected, and the known distance was set to 200 um. For future images, the "Disable Global Calibration" was unchecked when the images were opened.

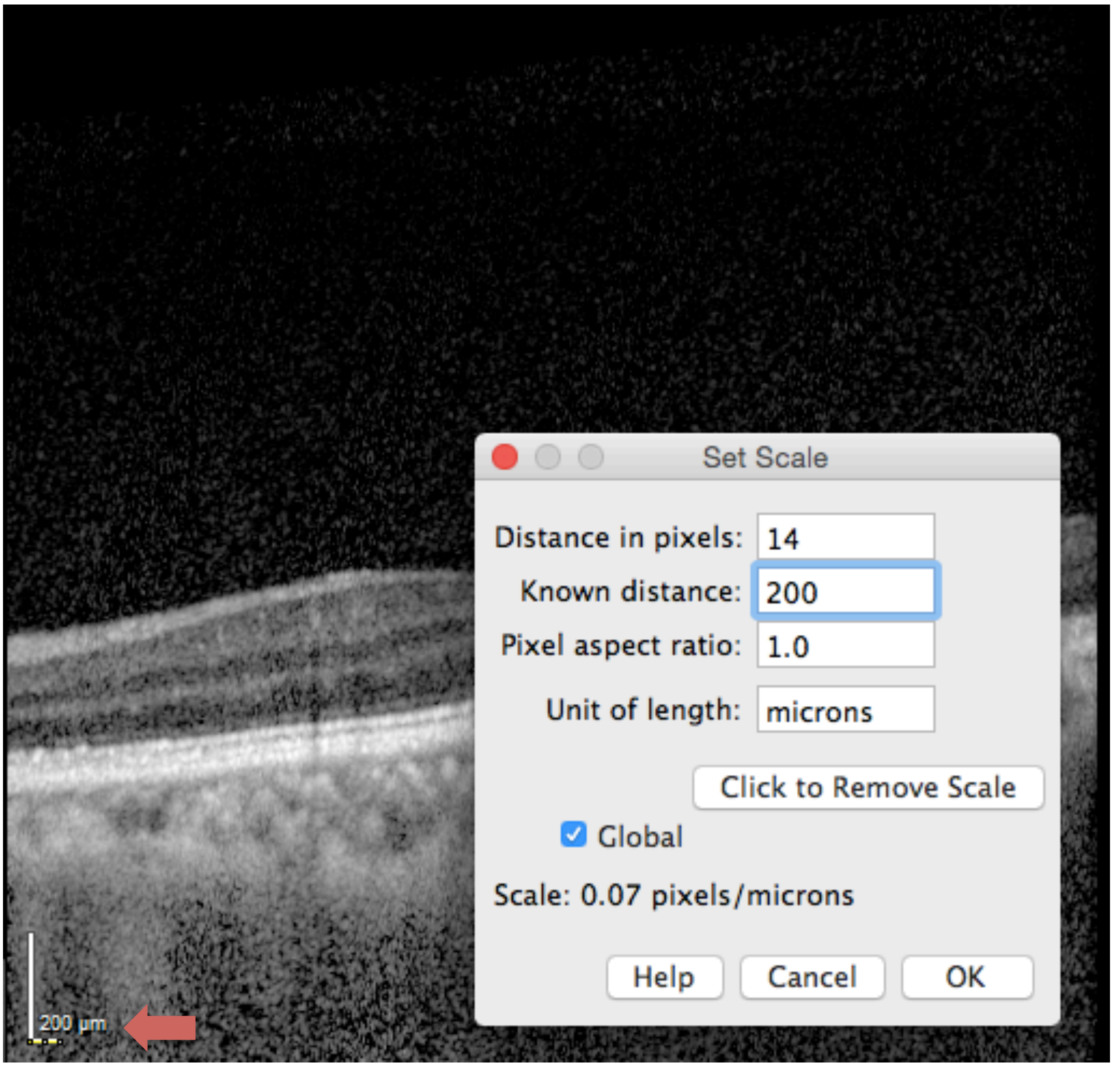

Fig. 1

Secondly, 2 750 um lines were drawn above the fovea, from the center of the fovea. The lines were drawn by first using the Colour Picker tool to select white, then, using the line tool, 750 um was measured parallel to the retinal pigment epithelium and drawn from the centre of the fovea, above the retina. "Command-D" was keyed in to leave a mark where the line was initially drawn.

Next, the polygon tool was used to draw a polygon under the lines drawn, to select the subfoveal choroid (Fig. 2). The area of the choroid under the retinal pigement epithlium was selected. The points of the polygon corresponded to the tips of the choroidal vessels, as represented by the hyporeflective areas. The area

was saved in the ROI manager by selecting the option Analyse > Tools > ROI manager, and Add [t] (Fig. 3).

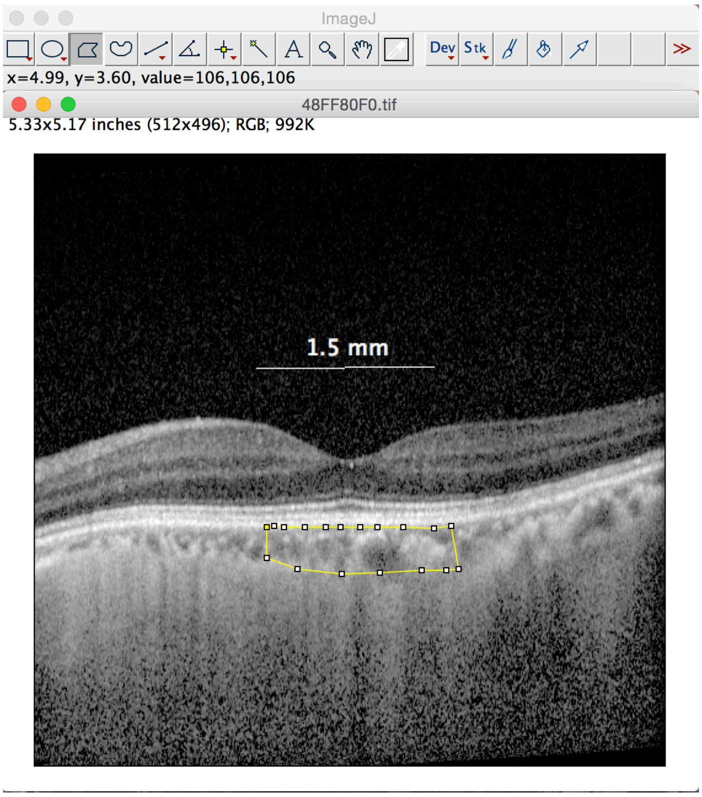

Fig. 2

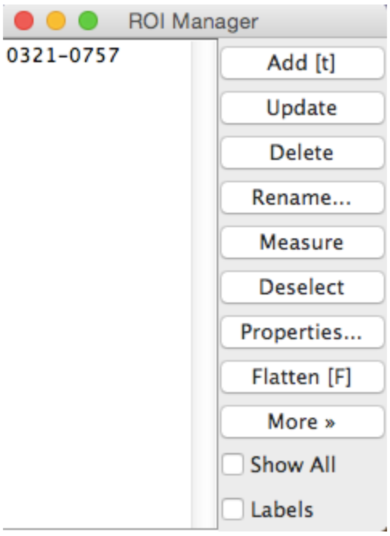

Fig. 3

Fourthly, auto threshold was applied. Image > Type > 8 bit was first applied (Fig. 4), followed by Image > Adjust > Auto Local Threshold (Fig. 5). Niblack was selected as the method (Fig. 6).

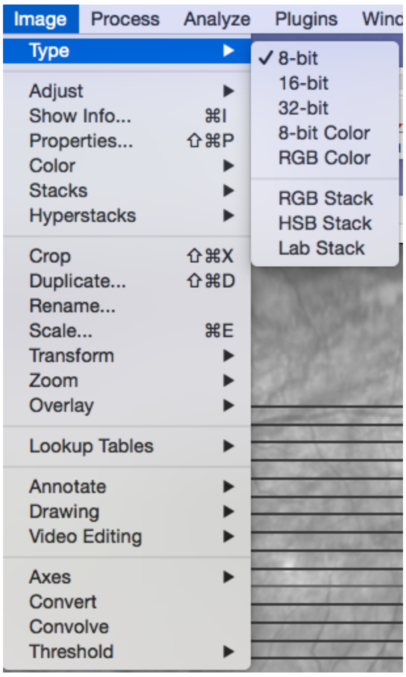

Fig. 4

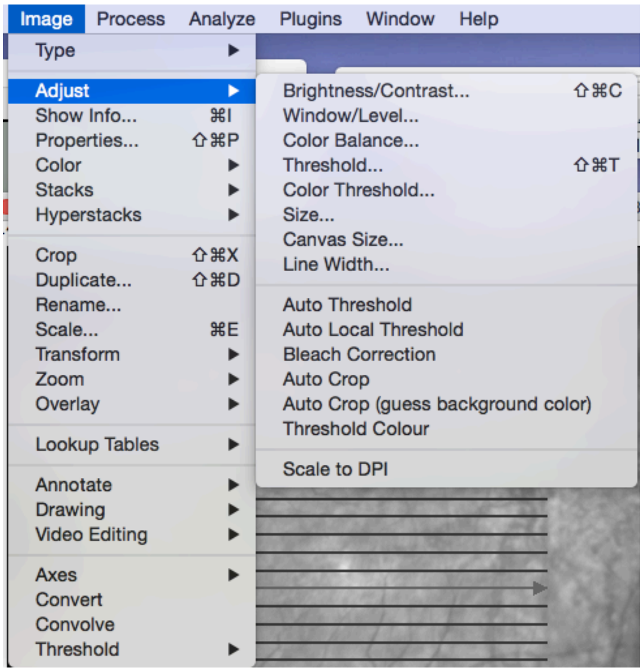

Fig. 5

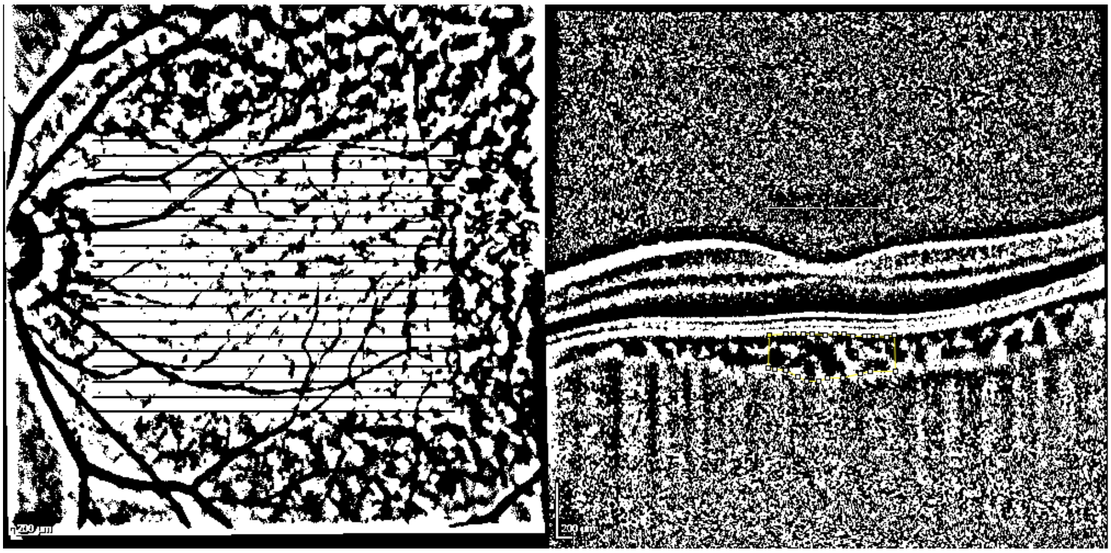

Fig. 6

Fifthly, the area of vascularity was highlighted by selecting Image > Type > RGB colour, and Image > Adjust > Colour Threshold. The first bar under Brightness was adjusted to 0, and the second bar was adjusted to 254 (Fig. 7). After clicking Select (Fig. 8), the area was added to the ROI manager by selecting Add [t]. to determine the area of vascularity within the initially selected polygon, both areas in the ROI manager were selected (Fig. 9) and merged by selecting More > AND (Fig. 10). The composite third area was added to the ROI manager by selecting Add [t] (Fig. 11).

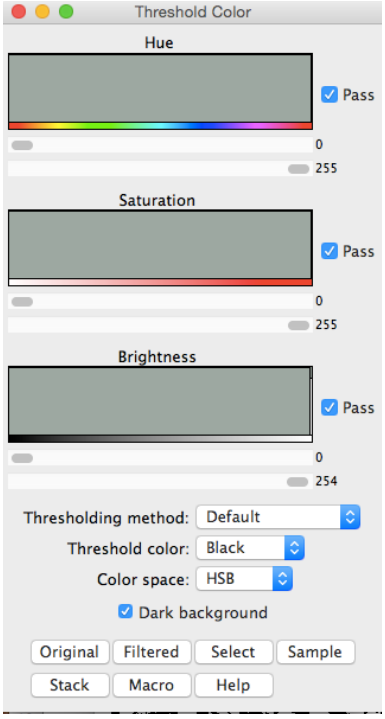

Fig. 7

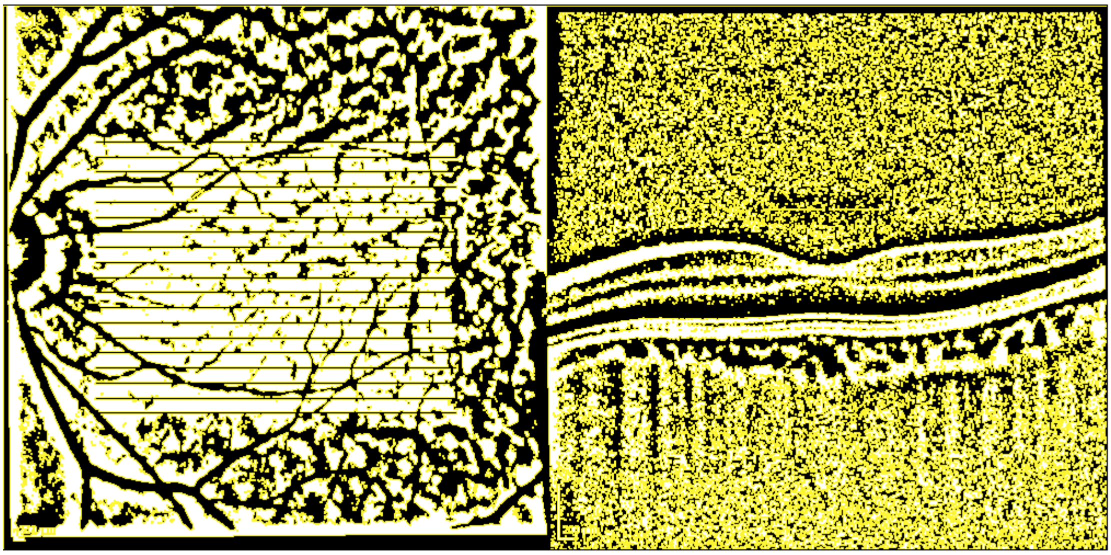

Fig. 8

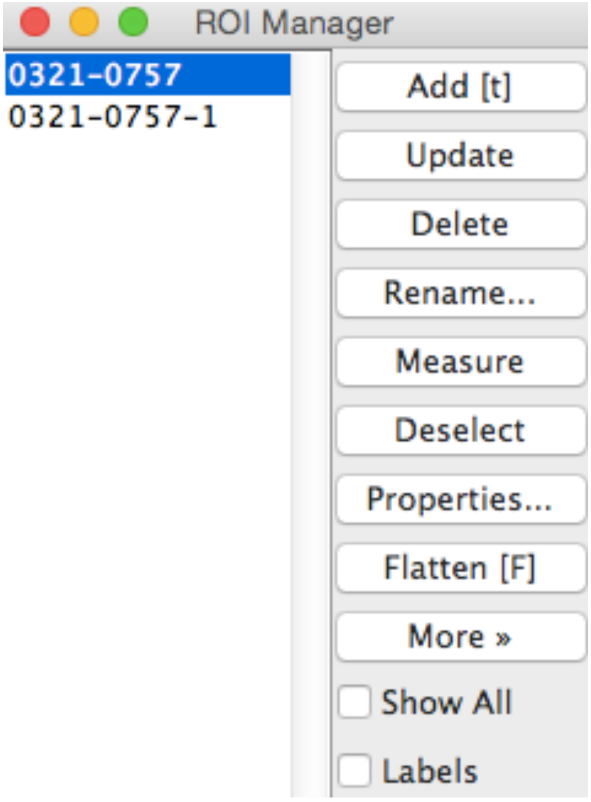

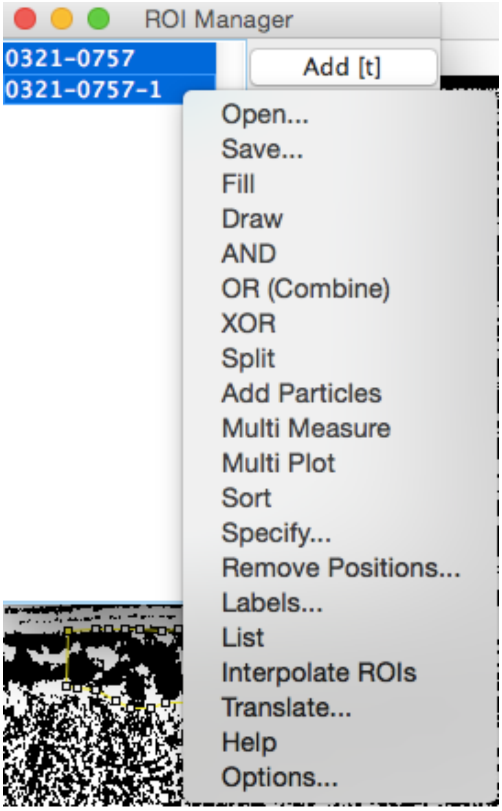

Fig. 9 Fig. 10

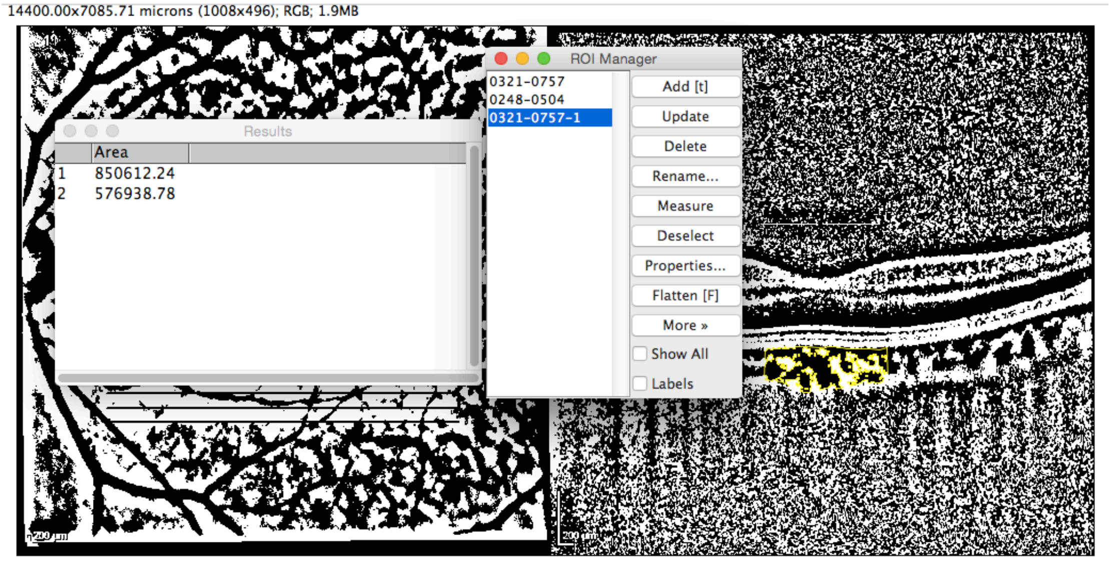

Fig. 11

Lastly, the first and third areas were selected and measured using the ROI manager. The first area represents the total area of choroid selected, and the third composite area is the vascular area.
